# Correction: Jensen et al. Prior Antibiotic Use Increases Risk of Urinary Tract Infections Caused by Resistant *Escherichia coli* among Elderly in Primary Care: A Case-Control Study. *Antibiotics* 2022, *11*, 1382

**DOI:** 10.3390/antibiotics12020386

**Published:** 2023-02-14

**Authors:** Maria L. V. Jensen, Volkert Siersma, Lillian M. Søes, Dagny Nicolaisdottir, Lars Bjerrum, Barbara J. Holzknecht

**Affiliations:** 1 Department of Clinical Microbiology, Copenhagen University Hospital—Herlev and Gentofte, 2730 Herlev, Denmark; 2 The Research Unit for General Practice and Section of General Practice, Department of Public Health, University of Copenhagen, 1014 Copenhagen, Denmark; 3 Department of Clinical Microbiology, Copenhagen University Hospital—Hvidovre and Amager, 2650 Hvidovre, Denmark; 4 Department of Clinical Medicine, University of Copenhagen, 2200 Copenhagen, Denmark

## Error in Figure/Table

In the original publication [1], there was a mistake in Table 2 “Adjusted analyses of total exposure to antibiotics (number of prescriptions and DDD) and time since last prescription, and OR of resistant *E. coli*” as published. The last row of the table is wrongly titled, and the presented results in this row are not correct. The corrected Table 2 appears below. The authors state that the scientific conclusions are unaffected. This correction was approved by the Academic Editor. The original publication has also been updated.

## Figures and Tables

**Table 2:** Adjusted analyses of total exposure to antibiotics (number of prescriptions and DDD) and time since last prescription, and OR of resistant *E. coli*.

		Mecillinam		Trimethoprim		Nitrofurantoin		Multi-Resistance	
		OR *	CI95% **	OR	CI95%	OR	CI95%	OR	CI95%
Number of prescriptions	0	Ref		Ref		Ref		Ref	
	1	1.32	(1.15;1.53)	1.40	(1.32;1.50)	1.25	(0.97;1.61)	1.35	(0.59;3.08)
	2	1.82	(1.59;2.07)	1.66	(1.56;1.77)	2.10	(1.69;2.60)	2.86	(1.45;5.63)
	≥3	2.47	(2.18;2.80)	3.11	(2.92;3.31)	4.53	(3.76;5.46)	11.53	(6.37;20.85)
Number of DDD ***	0	Ref		Ref		Ref		Ref	
	>0–33.3 percentile	1.56	(1.39;1.76)	1.54	(1.46;1.63)	1.84	(1.51;2.23)	1.34	(0.59;3.05)
	>33.3–6.66 percentile	1.88	(1.63;2.18)	2.03	(1.89;2.17)	2.59	(2.07;3.24)	2.29	(1.07;4.95)
	>66.6 percentile	2.60	(2.26;2.99)	3.22	(2.99;3.46)	4.49	(3.65;5.51)	10.21	(5.78;18.02)
Time since last prescription	No exposure	Ref		Ref		Ref		Ref	
	8–30 days	2.13	(1.91;2.38)	2.20	(2.09;2.32)	2.83	(2.38;3.37)	5.81	(3.37;10.03)
	31–60 days	1.53	(1.32;1.78)	1.71	(1.60;1.83)	2.51	(2.00;3.14)	3.72	(1.93;7.16)
	61–90 days	1.51	(1.27;1.79)	1.49	(1.38;1.62)	1.81	(1.37;2.40)	2.15	(0.85;5.40)

* OR = Odds Ratio. ** CI95% = 95% Confidence interval. *** For the different resistance patterns, the 33.3/66.6 percentiles were as follows: mecillinam 10/21.88, trimethoprim 10/21.88, nitrofurantoin 10.5/22.00, multi-resistance 10/18 DDD.

## References

[1] Jensen M.L.V., Siersma V., Søes L.M., Nicolaisdottir D., Bjerrum L., Holzknecht B.J. (2022). Prior Antibiotic Use Increases Risk of Urinary Tract Infections Caused by Resistant *Escherichia coli* among Elderly in Primary Care: A Case-Control Study. Antibiotics.

