# Supplementary material for: Prior Antibiotic Use Increases Risk of Urinary Tract Infections Caused by Resistant Escherichia coli among Elderly in Primary Care: A Case-Control Study
Source: Antibiotics (Basel). 2022 Oct 9;11(10):1382. doi: 10.3390/antibiotics11101382 (PMC9598065; doi:10.3390/antibiotics11101382)
Supplement: Supplementary file 1 [file antibiotics-11-01382-s001.zip › antibiotics-1912772-supplementary.pdf]

Supplementary Table S1: Infectious disease related ICD10 codes

DA39: Infections with meningococci

DA40: Sepsis caused by streptococci

DA41: Sepsis, other

DA49: Bacterial infection, without specification of location

DB35: Skin fungus

DB36: Other superficial fungi infection

DB37: Infection caused by candida albicans

DB38: Coccidioidomycosis

DB39: Histoplasmosis

DB40: Blastomycosis

DB42: Sporotricosis

DB44: Aspergillosis

DB45: Kryptococcsis

DB46: Zygomycosis

DB48: Mycosis, other

DB49: non-specificed mycosis

DG00: Bacterial meningitis

DG01: Meningitis in bacterial infections classified otherwise

DG06: Intra craniel and intra spinal abcesses and granulomas

DH65: Otitis media, non-purulent

DH66: Otitis media, purulent

DH67: Otitis media, classified otherwise

DJ00: Common cold

DJ01: Sinuitis

DJ02: Pharyngitis

DJ03: Sore throat

DJ04: Acute laryngitis

DJ05: Pseudocroup and acute epiglottitis

DJ06: acute laryngo-pharyngitis

DJ09: Influenza

DJ10: Influenza

DJ11: Influenza

DJ12: Pneumonia, viral

DJ13: Pneumonia, pneumococcus

DJ14: Pneumonia, Haemophilius

DJ15: Pneumonia, other bacterial

DJ16: Pneumonia, other infectious agent

DJ17: Pneumonia, classified otherwise

DJ18: Influenza

DJ36: Abscess, thorat

DJ40: Bronchitis

DJ41: Bronchitis, chronic

DJ42: Bronchitis, chronic otherwise classified

DJ44: Chronic obstructive pulmonary disease

DL00: Dematitis, caused by staphylococcus

DL01: Impetigo

DL02: Abscess, skin

DL03: Phlegmone

DL04: Acute lymadenitis

DL05: Pilonidal cyst

DL08: Infection located to the skin and subcutaneous tissue

DN30: Cystitis

DN34: Urinary tract infection

DN42: Other disease relation to the prostate

DN45: Epididymitis

DN49: Other infections related to male reproductive organs

DN70: Infection in uterine tube and ovaries

DN71: Uterine infections, except cervisitis

DN72: Cervicitis

DN73: Other form of infection related to female reproductive organs

DN75: Disease in Bartholin's gland

DN76: Infections in vagina and the outer female reproductive organs

DN77: Wounds and infections the outer female reproductive organs

Supplementary Table S2: Unadjusted analyses results

	Mecillinam		Trimethoprim		Nitrofurantoin		Multiresistance	
Exposure	c_OR*	CI95%**	c_OR	CI95%	C_OR	CI95%	c_OR	CI95%
Number of prescriptions								
0								
1	1.37	(1.19;1.58)	1.41	(1.32;1.51)	1.29	(1.01;1.66)	1.40	(0.62;3.19)
2	1.91	(1.68;2.18)	1.67	(1.57;1.78)	2.16	(1.74;2.69)	2.93	(1.50;5.72)
≥3	2.73	(2.42;3.08)	3.12	(2.93;3.32)	4.73	(3.93;5.69)	12.08	(6.93;21.06)
Number of DDD								
0								
>0-33.3 percentile***	1.62	(1.44;1.82)	1.55	(1.47;1.63)	1.88	(1.55;2.29)	1.78	(0.91;3.50)
33.3-66.6 percentile	2.02	(1.75;2.33)	2.04	(1.91;2.19)	2.69	(2.15;3.37)	5.91	(3.1;11.29)
>66.6 percentile	2.91	(2.54;3.33)	3.24	(3.02;3.48)	4.70	(3.83;5.78)	14.37	(7.96;25.95)
Time since last prescription								
No exposure	Ref		Ref				Ref	
8–30 days	2.29	(2.06;2.55)	2.23	(2.12;2.35)	2.95	(2.48;3.5)	6.23	(3.65;10.64)
31–60 days	1.64	(1.42;1.90)	1.73	(1.62;1.86)	2.61	(2.08;3.27)	4.04	(2.10;7.77)
61–90 days	1.60	(1.35;1.89)	1.51	(1.39;1.63)	1.87	(1.40;2.48)	2.32	(0.92;5.86)
Exposure to specific drugs within 90 days (Ref: no exposure)								
Phenoxymethylpenicillin or dicloxacillin	2.14	(1.86;2.45)	1.21	(1.11;1.31)	1.30	(1.02;1.65)	1.12	(0.49;2.57)
Pivmecillinam	1.75	(1.58;1.94)	1.39	(1.32;1.47)	1.46	(1.23;1.74)	3.55	(2.30;5.48)
Amoxicillin	2.05	(1.64;2.57)	1.32	(1.16;1.50)	0.85	(0.50;1.45)	-****	-
Amoxicillin + β lactamase inhibitor	2.01	(1.43;2.80)	1.35	(1.12;1.64)	1.54	(0.88;2.69)	10.24	(4.51;23.24)
Trimethoprim	1.50	(1.26;1.80)	6.52	(5.97;7.13)	1.10	(0.73;1.66)	9.99	(5.48;18.18)

Sulfamethizole	1.27	(1.08;1.48)	1.42	(1.32;1.54)	1.06	(0.80;1.4)	1.44	(0.72;2.90)
Nitrofurantoin	1.65	(1.40;1.95)	1.88	(1.73;2.05)	8.80	(7.35;10.52)	9.92	(6.07;16.22)
Macrolides	0.89	(0.66;1.21)	1.20	(1.05;1.37)	1.05	(0.69;1.6)	-	-
Quinolones	1.07	(0.81;1.42)	2.22	(1.98;2.48)	1.81	(1.23;2.65)	3.26	(1.23;8.58)
Others	1.95	(1.65;2.30)	1.59	(1.45;1.74)	1.26	(0.89;1.78)	3.34	(1.76;6.36)

* c_OR= crude odds ratio

** CI95%= 95% Confidence interval

*** For the different resistance patterns, the 33,3/66,6 percentiles were as following: mecillinam 10/21.88, trimethoprim 10/21.88, nitrofurantoin 10.5/22.00, multiresistance 10/18
**** no analysis due to low numbers

Supplementary Table S3: Sensitivity analyses results, adjusted.

		MECILLINAM			TRIMETHOPRIM			NITROFURANTOIN			MULTIRESISTANCE		
		OR	CI95%		OR	CI95%		OR	CI95%		OR	CI95%	
**NUMBER OF PRESCRIPTIONS**													
	0	Ref			Ref			Ref			Ref		
	1	1.35	1.13	1.61	1.50	1.39	1.63	1.30	0.94	1.79	1.73	0.54	5.59
	2	1.90	1.61	2.24	1.63	1.50	1.78	1.83	1.36	2.46	3.66	1.36	9.82
	3+	2.34	2.01	2.73	3.57	3.31	3.85	5.98	4.81	7.43	12.12	5.22	28.11
**NUMBER OF DDD**													
	0	Ref			Ref			Ref			Ref		
	>0-33.3 percentile	1.61	1.39	1.86	1.63	1.52	1.75	1.94	1.52	2.49	2.07	0.77	5.58
	>33.3-66.6 percentile	1.83	1.53	2.20	2.21	2.03	2.41	3.02	2.31	3.95	6.29	2.45	16.12
	>66.6 percentile	2.54	2.14	3.01	3.68	3.36	4.03	5.84	4.59	7.43	14.33	6.02	34.12
**TIME SINCE LAST PRESCRIPTION**													
	No exposure*	Ref			Ref			Ref			Ref		
	8 days–30 days	2.10	1.83	2.40	2.39	2.24	2.55	3.42	2.76	4.23	6.62	3.02	14.49
	>30 days–60 days	1.60	1.33	1.92	1.86	1.71	2.03	2.82	2.15	3.70	3.05	1.01	9.22

	>60 days–90 days	1.44	1.14	1.81	1.59	1.43	1.77	1.93	1.34	2.76	3.56	1.08	11.73
**PHENOXYMETHYLPENICILLIN OR DICLOXACILLIN**													
	No	Ref			Ref			Ref			Ref		
	Yes	2.16	1.83	2.54	1.20	1.09	1.32	1.29	0.96	1.73	1.36	0.49	3.78
**PIVMECILLINAM**													
	No	Ref			Ref			Ref			Ref		
	Yes	1.58	1.39	1.80	1.53	1.43	1.63	1.69	1.40	2.05	4.22	2.23	7.96
**AMOXICILLIN**													
	No	Ref			Ref			Ref					
	Yes	1.74	1.32	2.30	1.29	1.10	1.52	0.56	0.28	1.14			
**AMOXICILLIN + B LACTAMASE INHIBITOR**													
	No	Ref			Ref			Ref			Ref		
	Yes	1.77	1.18	2.65	1.45	1.15	1.84	1.07	0.51	2.26	5.19	1.20	22.40
**TRIMETHOPRIM**													
	No	Ref			Ref			Ref			Ref		
	Yes	1.43	1.13	1.80	7.19	6.38	8.11	1.81	1.34	2.46	6.17	2.36	16.16
**SULFAMETHIZOLE**													
	No	Ref			Ref			Ref			Ref		
	Yes	1.15	0.94	1.42	1.46	1.32	1.61	1.19	0.88	1.61	1.44	0.51	4.07
**NITROFURANTOIN**													
	No	Ref			Ref			Ref			Ref		
	Yes	1.65	1.35	2.03	2.48	2.23	2.76	11.77	9.70	14.29	13.44	6.75	26.75
**MACROLIDES**													
	No	Ref			Ref			Ref					
	Yes	0.80	0.55	1.17	1.21	1.03	1.42	0.75	0.40	1.42			
**QUINOLONES**													
	No	Ref			Ref			Ref			Ref		
	Yes	1.00	0.72	1.38	3.13	2.71	3.62	2.51	1.79	3.51	2.29	0.55	9.52
**OTHERS**													
	No	Ref			Ref			Ref			Ref		
	Yes	1.66	1.35	2.05	1.68	1.50	1.88	1.35	0.96	1.89	3.75	1.56	9.04
